# Unravelling the demographic dynamics of ethnic residential segregation

**DOI:** 10.1002/psp.2193

**Published:** 2018-08-24

**Authors:** Timo M. Kauppinen, Maarten van Ham

**Affiliations:** ^1^ Social Policy Research Unit National Institute for Health and Welfare Helsinki Finland; ^2^ OTB—Research for the Built Environment Delft University of Technology Delft The Netherlands; ^3^ School of Geography and Sustainable Development University of St Andrews St Andrews UK

**Keywords:** decomposition, ethnic segregation, Finland, immigrants, population dynamics

## Abstract

Selective intraurban migration of ethnic groups is often assumed to be the main microlevel mechanism reproducing ethnic residential segregation. However, other demographic processes, such as natural change and international migration, also matter. This paper contributes to the literature by unravelling the impacts of different demographic processes to changes in ethnic segregation. It uses longitudinal individual‐level register data on the complete population of the Helsinki region in Finland. We calculate observed changes in exposure indices, segregation indices in counterfactual scenarios, and decompositions of population changes. Results indicate that intraregional migration is the main process affecting segregation between Finnish‐origin and non‐Western‐origin populations, but whereas migration of the former increases segregation, migration of the latter decreases it. International migration and natural change among the non‐Western‐origin population are the main processes increasing exposure of the non‐Western‐origin population to other members of the group. No indication is found of a general tendency to “self‐segregate.”

## 1 INTRODUCTION

Ethnic residential segregation is often seen as problematic because it is thought to hinder integration, particularly if segregation is a consequence of the self‐segregation of immigrants. As van Gent and Musterd (2016, pp. 894–895) put it:

Consequently, local authorities may continue to regard high levels of social spatial segregation and migrant concentrations as problematic, leading to calls for “social integration” and for the “integration of migrants” … yet it is unsure what patterns of social and ethnic segregation are emergent.

The implications of residential segregation depend on what mechanisms produce it. In addition to macrolevel structural factors and the historical legacies of the local context (Musterd, Marcinczak, van Ham, & Tammaru, 2017), microlevel processes are also important. In the case of ethnic segregation, selective intraurban migration of different ethnic groups between neighbourhoods is often assumed to be the main mechanism. Politically, this is also the most salient mechanism, as it may indicate preferences for coethnic neighbours or constraints regarding spatial integration (e.g., Boschman & van Ham, 2015). On the other hand, if intraurban migration of an ethnic group does not contribute to increasing segregation, there are less grounds to assume that a tendency for self‐segregation exists.

However, other demographic processes, such as natural population change and international migration, also contribute to changes in ethnic segregation. Positive natural change (excess number of births over deaths) among immigrants has been found to be an important contributor to the growth of ethnic minority concentrations in several European contexts (Bråmå, 2008; Finney & Simpson, 2009; Wessel, Magnusson Turner, & Nordvik, 2018; Zwiers, van Ham, & Manley, 2017), and the contributions of natural change and immigration may be even more important when larger scale immigration is relatively recent. Finland is an example of such a context, with increasing immigration only since the 1990s. There are indications that these two processes have an important role in the Helsinki region (Vilkama, 2011), warranting more detailed analyses of the different components driving segregation. If the development of ethnic segregation is driven to a considerable extent by immigration and natural growth, the implications for policy differ from segregation driven by intraurban migration.

Earlier studies on the effects of different demographic processes on the development of ethnic segregation have usually investigated only the effects of a limited set of demographic processes. They have also typically used only one measure of segregation, while alternative measures can lead to different outcomes. Many studies have focused on comparing changes occurring in different types of neighbourhoods instead of characterising the direction of change of the whole urban region, and an analysis of the majority ethnic group has not always been included in the studies.

Our study responds to the call by Sampson and Sharkey (2008) to study the aggregate consequences of individual‐level neighbourhood change processes. In doing so, we investigate the effect of several demographic processes at the same time. By analysing two dimensions of segregation—evenness and exposure—we provide insights into the significance of different demographic processes for the development of segregation within a region, instead of focusing only on particular (types of) neighbourhoods. Furthermore, and of critical importance, we analyse demographic processes not only among the immigrant‐origin population but also among the native‐origin population. Demographic processes among the native‐origin population can also affect segregation.

The aim of this paper is to provide more insights into the contributions of different demographic processes on the dynamics of ethnic residential segregation. We use longitudinal, individual‐level, register‐based data on the whole population of Finland to assess this question for the Helsinki region using three different methodological approaches. First, we use a counterfactual method in which the significance of each population‐change process is assessed by comparing observed segregation to a counterfactual scenario omitting this population‐change process. Here, we apply a method previously used by Finney and Simpson (2009), Bailey (2012), and Bailey, van Gent, and Musterd (2016). In addition to the counterfactual method, we apply two other methods: an analysis of the observed changes in exposure to non‐Western immigrants and the decomposition of population changes in different types of neighbourhoods. Together, these three approaches will offer greater insight into how different demographic processes are contributing to ethnic segregation dynamics.

## 2 EARLIER STUDIES

### 2.1 Demographic approach to the analysis of residential segregation

Finney and Simpson (2009) contend that a demographic approach is essential for understanding the development of ethnic residential segregation. In addition to migration, natural change (births and deaths) must be analysed as a contributory process. The roles of different demographic processes may differ between ethnic groups depending on their age structure and time and type of immigration. Such demographic analysis may bring new and important insights regarding the causes of segregation.

Among the ethnic minority population that is already living in an urban region, intraurban migration can be expected to be the main mechanism affecting segregation (Boschman & van Ham, 2015). Intraurban migration is also related to the idea of “self‐segregation,” that is, the voluntary residential mobility of ethnic minorities into neighbourhoods with higher shares of ethnic minorities. A preference for coethnic neighbours might lead to higher in‐migration of ethnic minorities into neighbourhoods with ethnic minority concentrations as compared with out‐migration from them. However, this could also be a sign of low economic resources among immigrants or of such constraints as discrimination on the housing market or of expected discrimination in other neighbourhoods (for an overview of the main theoretical frameworks, see,e.g., Bolt, van Kempen, & van Ham, 2008). Similarly, among the majority ethnic group, an excess of out‐migration from these minority concentration neighbourhoods might indicate avoidance or “flight” behaviour (Bråmå, 2006).

In order to differentiate between the effects of different demographic processes on ethnic segregation, the population changes of an ethnic group in an area must be decomposed into changes via natural change (births–deaths) and migration (arrivals–departures) (Finney & Simpson, 2009). Therefore, at least four components of population change have to be measured: births, deaths, in‐migration, and out‐migration. Migration needs to be further subdivided into at least the categories local (i.e., intraregional) and nonlocal (international and between‐region) migration. When applying Bailey's (2012) “neighbourhood accounts” framework, deaths, out‐migration from the region, and also moves to the nonhousehold population (such as moves to institutions) should be considered as processes of exit from the household population of the area. Similarly, intraregional migration can be seen as a process of change within the “core” group (those belonging to the household population of the area at the beginning and at the end of the study period); and births, in‐migration to the region, and moves from the nonhousehold population can be seen as points of entry to the household population.

### 2.2 Descriptive findings from earlier studies

European studies analysing the population changes of ethnic groups in ethnic minority concentration areas have found that natural change can be an important factor in increasing the ethnic minority populations in such areas (Bråmå, 2008; Finney & Simpson, 2009; Musterd & de Vos, 2007; Wessel et al., 2018; Zwiers et al., 2017). Also, nonlocal migration (Bråmå, 2008), and especially international migration (Wessel et al., 2018), has been found to increase the ethnic minority populations in these areas.

In two Nordic studies, the general direction of intraurban migration of minority ethnic groups has been found to be mostly away from minority concentrations towards native‐origin communities in Gothenburg, Sweden (Bråmå, 2008), and in Oslo, Norway (Wessel et al., 2018). Similar findings have been obtained from the Netherlands (Musterd & de Vos, 2007; Zwiers et al., 2017), although these studies have not differentiated between different types of residential mobility, studying instead total residential mobility. In an earlier Finnish study, Vilkama (2011) found the intraurban migration of ethnic minorities to have a slightly concentrating tendency.

Some studies have also analysed the intraurban migration of the *native‐origin* population. Bråmå (2008) and Vilkama (2011) found it to be directed away from minority concentrations. Musterd and de Vos (2007) had similar findings for the Netherlands regarding *total* residential mobility of the native Dutch population, but according to Zwiers et al. (2017), the migration of the ethnic majority has become directed towards the ethnic minority concentrations in more recent years, potentially due to urban restructuring.

### 2.3 Counterfactual designs

Previous studies analysing the contributions of different sociodemographic processes either to ethnic or to socio‐economic segregation have mostly analysed the contributions of different processes to population changes in particular neighbourhoods or types of neighbourhoods, especially ethnic minority concentrations. Some studies have also constructed counterfactual scenarios that either omit a particular population‐change process or allow only one process to occur, and then these studies compared the observed development of segregation indices with values obtained in these counterfactual scenarios. The advantage of these counterfactual designs is that they aim to characterise the contributions of different processes at an aggregate level, for example, of urban regions, instead of focusing on individual neighbourhoods.

In order to investigate the effects of different processes at the aggregate level, some studies have been designed in such a way that the effect of one demographic process at a time is removed, and the resulting segregation indices are compared with actually observed results. Finney and Simpson (2009) used the isolation index (P*), which measures the extent to which minority residents are exposed to each other (Massey & Denton, 1988). They estimated a change of P* for each ethnic group with and without natural change among the group during the study period. At the end of the period, the index was calculated for two populations: (a) the observed population and (b) the population as it would have been without the effect of natural change over the same period. The difference between these values indicated the impact of natural change. Wessel et al. (2018) studied the effects of several demographic processes using this approach, and they applied both the isolation index and the index of dissimilarity (*D*), which measures the dissimilarity in the residential distributions of two groups (e.g., White, 1983).

Other studies have removed the effects of all other processes while studying the effect of one process. Bailey (2012) assessed the contribution of each flow (population change component) to changes in socio‐economic segregation by looking at *D* “before” and “after” each flow occurred. For example, the combined effect of “exit” flows was calculated as the difference between the observed segregation in the “core” group before the flows and segregation in the same group when all persons exiting the sample had been removed. Bailey (2012) notes that the sum of the effects of individual processes of change on *D* may not be the same as the observed total change, as the different changes overlap with each other. Additionally, unlike the exposure indices (Quillian, 2012), *D* cannot be decomposed additively into the contributions of subgroups, so the individual effects may not be expected to sum up perfectly to the observed total change.

The choice of the segregation index used to study the effect of different demographic processes is important. For instance, P* is more sensitive than *D* to changes in the size of an ethnic group (e.g., Massey & Denton, 1988). Therefore, P* may be particularly strongly affected by natural change and immigration.

Studies applying counterfactual designs have suggested that natural change accounted for the majority of the increase in P* at the district level among the Indian, Pakistani, and Bangladeshi groups in Britain between 1991 and 2001 (Finney & Simpson, 2009), and that it was important also for the increase in P* among the non‐Nordic population in Oslo, Norway (Wessel et al., 2018). Finney and Simpson (2009) combined this finding with information on dispersing internal migration, concluding that the rest of the increase in P* is mostly due to immigration and that arguments of “divisive” segregation based on the development of P* should be questioned. If increases in P* are not based on internal migration of ethnic minorities to minority concentrations, then self‐segregation does not seem to be an important explanation for the increase.

Wessel et al. (2018) used the same design to analyse the contributions of other demographic processes as well, using both P* (exposure) and *D* (dissimilarity) as segregation measures. They likewise found that the immigration of non‐Nordic immigrants increases P*. The finding of natural change contributing to increasing segregation persisted when using *D*, but in contrast to the results obtained with P*, international migration decreased segregation when measured with *D*. Against their expectations—and the descriptive analysis of population changes—they did not find clear effects of intraurban migration for non‐Nordic immigrants with either measure. Neither did they find effects of migration between municipalities. These findings demonstrate that at the aggregate regional level, the effects of the demographic processes can be different as compared with analysing only concentrations of ethnic minorities. Furthermore, processes increasing the exposure of immigrants to each other may nevertheless decrease the residential separation between the ethnic minorities and the ethnic majority. However, the contribution of demographic processes within the ethnic majority was not assessed by Wessel et al. (2018).

In the case of *socio‐economic* segregation (by income or occupational status), Bailey (2012) found selective migration to have only a minor role in explaining the changes in socio‐economic segregation in Scotland between 1991 and 2001, whereas social mobility had a much greater impact. On the other hand, in a comparison between Amsterdam and The Hague, Bailey et al. (2016) found significant differences between the cities in terms of the contribution of different processes of change to the development of income segregation. In Amsterdam, changes in overall segregation were mainly driven by intraurban residential mobility and in‐migration, whereas in The Hague, the changes were mostly driven by in‐migration.

### 2.4 Approach of this paper

In this paper, we contribute to the literature by combining several approaches that have previously been applied in separate studies, some of which have addressed socio‐economic segregation instead of ethnic segregation. We analyse changes in ethnic segregation with a similar counterfactual method as used by Finney and Simpson (2009) and Wessel et al. (2018). We analyse the contributions of multiple demographic processes of change, similar to Bailey (2012), Bailey et al. (2016), and Wessel et al. (2018), and we use both isolation and dissimilarity indices to measure segregation (as Wessel et al., 2018). Unlike earlier studies that apply the counterfactual design to assess ethnic segregation dynamics, we also study the demographic dynamics within the native‐origin population. We complement the counterfactual analysis by decomposing population changes in different types of neighbourhoods and by looking at the observed changes in exposure to the ethnic minority.

The relatively low levels of income inequality in Finland and of residential segregation in the Helsinki region (Skifter Andersen, Andersson, Wessel, & Vilkama, 2016; Vaattovaara, Vilkama, Yousfi, Dhalmann, & Kauppinen, 2010) are factors that reduce barriers to mobility between neighbourhoods (Nieuwenhuis, Tammaru, Ham, Hedman, & Manley, 2017). On the other hand, they can also lead to less need for spatial mobility (Wessel, Andersson, Kauppinen, & Skifter Andersen, 2017).

## 3 RESEARCH DESIGN

### 3.1 Data

We used longitudinal, register‐based, individual‐level data from Statistics Finland on the whole population of Finland for 2004–2014 (contract TK‐52‐1417‐16). The analysis focuses on the Helsinki region, defined as the “subregion” (*seutukunta*) around the capital city Helsinki, which approximates a travel‐to‐work area. This region represents the Local Administrative Unit 1 level in the Classification of Territorial Units for Statistics in the European Union (former NUTS 4 level). According to our data, in 2014, the population of the region was 1,483,000, with 219,023 (14.8%) people having an immigrant background (i.e., at least one foreign‐born parent). Altogether, 28% of the population of Finland and 50% of those with an immigrant background lived in this region in 2014. Using our data, we could track the research population annually between 2005 and 2014. The data contain crucial information on places of residence, non‐Western immigrant background, age, and deaths. We used zip‐code areas as the area units. The number of zip‐code areas in the Helsinki region (fixed to the 2015 delineation) was 303. The average population size within a particular zip‐code area in 2014 was 4,865 (*SD* = 4,741).

Ethnic categorisation was based on country‐of‐birth information. We defined immigrant origin as having at least one foreign‐born parent or, in the absence of parental information, being foreign‐born. As this definition is based only on countries of birth, naturalisation does not affect the measurement. We focused on a subgroup of the immigrant‐origin population that we call “non‐Western.” Non‐Western countries, in this case, refer to all non‐European countries except for the United States, Canada, Australia, and New Zealand. 1Analyses of more detailed groups are not done in order to keep the number of cases at a reasonable level. We categorised a person with an immigrant origin as belonging to a non‐Western‐origin group if the foreign‐born parent (or the person himself or herself in the case of missing parental information) was born in a non‐Western country. In the case of two foreign‐born parents, the mother's country of birth was prioritised.

The small size of the immigrant‐origin population in Finland, and, on the other hand, its fast growth, can be seen in the changing numbers of persons with a non‐Western background in the study region. The data show that whereas 39,363 persons with a non‐Western background lived in the Helsinki region in 2005, the number had increased to 83,401 by 2014 (112% growth). Corresponding numbers for the Finnish‐background population are 1.20 million and 1.24 million. 2The total population also includes those with other than a non‐Western immigrant background. The share of the non‐Western group out of the total immigrant‐background population increased in the study region from 38% in 2005 to 41% in 2014. Among the rest of the immigrant‐background population, those with an Estonian or Russian background are the largest groups. The share of the population having some immigrant background changed between 2005 and 2014 in Finland from 4.5% to 8.2% and in the Helsinki region from 8.3% to 14.8% (source: the dataset of the study) These numbers include people living in a household population in the region at either the beginning or the end of the corresponding periods (2005–2008, 2008–2011, 2011–2014; see Section 3.2), meaning that those living in the region but only in a nonhousehold population were excluded. The non‐Western group mostly originates from Africa and Asia (2014: 31% from North Africa or the Middle East, 14% from Somalia, 7% from China, 29% from other Asian countries, 14% from other African countries, and 6% from outside Africa or Asia, mainly Latin America). Somalia was the most common foreign country of birth (7%), followed by Iraq and China. The most common country of birth in the non‐Western‐origin group in 2014, however, was Finland, meaning that 35% of this group consisted of second‐generation immigrants.

### 3.2 Methodology

We studied three periods, 2005–2008, 2008–2011, and 2011–2014, as compared with conducting separate annual analyses, in order to increase the number of people in the analysis. Several shorter periods instead of one longer period of time were used because during a longer period of time, each individual may experience multiple different demographic events, making their categorisation more difficult in the analysis. The starting point of the analysis involved arranging the data into pairs of years, the beginning and end years of each period (*t*_0_ and *t*_1_), and categorising every person into a specific category measuring residential mobility or other demographic events occurring between those 2 years.

The “demographic balancing equation” (Finney & Simpson, 2009) shows how change in the population size of an ethnic group in a particular geographical area can be broken down into natural change and migration, each having two subcomponents:

$$\begin{array}{cc} \text{NATURAL CHANGE} & \text{MIGRATION}\\ \text{Population change of group}\;X=\left(\text{births},-,\text{deaths}\right)+\left(\text{arrivals},-,\text{departures}\right). \end{array}$$

In this study, arrivals and departures were further divided into intraregional, between‐region, and international components, whereas moves between a household and nonhousehold population were also measured. Household population refers to people living permanently in dwellings, so institutionalised people and those without permanent addresses in Finland were excluded. As a result, we arrived at the following categories: (a) those staying within the same zip‐code area (“stayers”), (b) intraregional migrants, (c) between‐region in‐migrants, (d) between‐region out‐migrants, (e) immigrants, (f) emigrants, (g) births, (h) deaths, (i) movers to the household population, and (j) movers from the household population. 3Some of those in the “from the household population” category may be emigrants whose emigration has not yet been observed in the population register. The more exact definitions of the categories are shown in Table 1.

**Table 1:** Definition of the population‐change categories

Population‐change category	In Finland at *t*_0_?	In Finland at *t*_1_?	In the region's household population at *t*_0_?	In the region's household population at *t*_1_?	Other criteria
Stayers	Yes	Yes	Yes	Yes	Same zip code in *t*_0_ and *t*_1_.
Intraregional movers	Yes	Yes	Yes	Yes	Different zip codes in *t*_0_ and *t*_1_.
Between‐region out‐movers	Yes	Yes	Yes		Did not emigrate from the region before moving to another region in Finland. Includes also those who first moved to another region in Finland before emigrating.
Between‐region in‐movers	Yes	Yes		Yes	Did not emigrate from other regions before immigrating to the region. Also includes all migrants to the region from other Finnish regions after *t*_0_ who were born between *t*_0_ and *t*_1_ outside the region (in Finland or elsewhere) and those who first immigrated to other regions in Finland and then moved to the region between *t*_0_ and *t*_1_.
Emigrants	Yes		Yes		Did not die while living in Finland between *t*_0_ and *t*_1_. Did not move to another region in Finland before emigrating. Includes also those who emigrated from the region before moving to another region in Finland.
Immigrants		Yes		Yes	Age over 0 years at *t*_1_. Did not first immigrate to other regions before moving to the region. Also includes those who first emigrated from other regions between *t*_0_ and *t*_1_ and then immigrated to the region.
Deaths	Yes		Yes		Died after *t*_0_, before or at *t*_1_, before any move out from the region (moves observed at the end of the year).
Births		Yes		Yes	Was living in the region aged 0 years at the end of some year after *t*_0_ and before or at *t*_1_.
From household population	Yes	Yes	Yes		In the region at *t*_1_, but not in the household population.
To household population	Yes	Yes		Yes	In the region at *t*_0_, but not in the household population.

Although certain demographic events may be connected with each other, in this study, they were treated as separate events. This concerns, for example, moves associated with a simultaneous or eventual birth in the mover's household. In this case, the move pertained to the mover and the birth to the child that was born. If someone moved and died during the same year, only the death was counted here, as the person could no longer be observed at the end of the year.

Next, the contributions of these demographic processes to changes in ethnic segregation, as measured by the segregation indices, were analysed. The index of dissimilarity (*D*) and exposure index (_*x*_ P*_y_) were used. The index of dissimilarity is the most common index used to measure the “evenness” dimension of residential segregation (Massey & Denton, 1988). It measures the residential separation between two groups (here: between the non‐Western‐origin population and the Finnish‐origin population). Its values can be interpreted as showing what share of either group should change their zip‐code area in order to have the same residential distribution in the two groups. It is not directly affected by changes in the overall share of immigrants in the population if the growth does not lead to a change in the immigrants' residential pattern. The exposure index, on the other hand, combines information on residential distribution with the share of immigrants in the population. As used here, the exposure index shows the average share of non‐Western‐origin population in the neighbourhoods of a particular “focal group” (the non‐Western‐origin population itself—in this case, it is called the isolation index—or some part of it, or the Finnish‐origin population), with neighbourhoods being weighted in the calculation by the proportion of the total focal group living in the neighbourhood. Therefore, the exposure index is not a pure segregation index, but its advantage is that it captures changes in the “visibility” of immigrants that are brought on simply by the increasing share of immigrants in the regional population.

The index of dissimilarity is calculated using the following formula in a two‐group situation (White, 1983):

$$D=\frac{1}{2}\sum_{i=1}^{n}\left|\left(\frac{x_{i}}{X}-\frac{y_{i}}{Y}\right)\right|.$$

The exposure index is calculated using the following formula (Massey & Denton, 1988):

$${}_{x}{P^{*}}_{y}=\sum_{i=1}^{n}\left[\left(\frac{x_{i}}{X}\right)\left(\frac{y_{i}}{t_{i}}\right)\right].$$

In both formulas, *i* refers to the zip‐code areas in a particular region, *x*_*i*_ to the population size of group *x* in zip‐code area *i*, *y*_*i*_ to the population size of group *y* in that area, *t*_*i*_ to the total population of a particular zip‐code area, *X* to the total population size of group *x* in the region, and *Y* to the total population size of group *y* in the region. When *x* and *y* are different groups, the exposure index is called the “interaction” index, and when they refer to the same group, the exposure index is called the “isolation” index.

The analysis proceeded in three steps, each applying a different method. We applied three methods because each of them has its shortcomings, while jointly, they give a full overview of changes in segregation and the contributions of the different demographic processes. The *first* step of the analysis is to focus on *observed* changes between *t*_0_ and *t*_1_. This is done by investigating the exposure of each population‐change category of either the non‐Western‐origin population or the Finnish‐origin population to the total non‐Western group, that is, the average percentage of non‐Western‐background population in their zip‐code areas at *t*_0_ and *t*_1_. These categories constitute the 10 categories mentioned above, based on different demographic processes of change between *t*_0_ and *t*_1_ (the beginning and end of each period). At *t*_0_, the following categories were observed: stayers, intraregional out‐migrants, between‐region out‐migrants, emigrants, deaths, and movers from a household population. Correspondingly, at *t*_1_, the following categories were observed: stayers, intraregional in‐migrants, between‐region in‐migrants, immigrants, births, and movers to the household population.

Change in the exposure values between *t*_0_ and *t*_1_ was then calculated within each broader population change process—no change, intraregional migration, between‐region migration, international migration, natural change, and moves to/from the household population. This was done by subtracting the exposure value of the population‐change category related to the process at *t*_0_ from the exposure value of the corresponding category at *t*_1_. In the case of stayers and intraregional movers, the values at *t*_0_ and *t*_1_ referred to the same persons, whereas, for example, in between‐region migration, the exposure value of eventual out‐movers at *t*_0_ was subtracted from the exposure value of in‐movers at *t*_1_. This shows how much higher the exposure to the non‐Western‐origin population was among the in‐movers at *t*_1_ than among the out‐movers at *t*_0_. Positive changes in exposure indicate that the in‐movers moved to a zip‐code area where their exposure to the non‐Western‐origin population was higher than the exposure of the out‐movers to the non‐Western‐origin population before they had moved. This design is illustrated in Table 2. The numbers of people with a non‐Western background in these categories are shown in Table A1.

**Table 2:** Calculation of the differences in exposure to the non‐Western‐origin population (Δ*x*_*i*_ *Py*) between categories representing each population change process at *t*_0_ and *t*_1_

Wider population change process	Population change category	When present	Exposure to the population with a non‐Western immigrant background		
			*x*_*i*_ *Py*(*t*_0_)	*x*_*i*_ *Py*(*t*_1_)	Δ*x*_*i*_ *Py*
No change	Stayers	*t*_0_ and *t*_1_	*P*_1_	*P*_2_	*P*_2_ − *P*_1_
Intraregional migration	Intraregional movers	*t*_0_ and *t*_1_	*P*_3_	*P*_4_	*P*_4_ − *P*_3_
Between‐region migration	Between‐region out‐movers	*t*_0_	*P*_5_		*P*_6_ − *P*_5_
	Between‐region in‐movers	*t*_1_		*P*_6_	
International migration	Emigrants	*t*_0_	*P*_7_		*P*_8_ − *P*_7_
	Immigrants	*t*_1_		*P*_8_	
Natural change	Deaths	*t*_0_	*P*_9_		*P*_10_ − *P*_9_
	Births	*t*_1_		*P*_10_	
Moves to/from household population	From household population	*t*_0_	*P*_11_		*P*_12_ − *P*_11_
	To household population	*t*_1_		*P*_12_	

The advantages of the method used in this first step are that we investigated changes that have actually been observed and the results are for the whole region instead of just parts of it. We were also able to observe stayers as well as movers. However, these results do not reveal the net effects of particular processes on the segregation levels, which depend also on the sizes of the groups in question at different time points. Another shortcoming is that we had to rely on the exposure index, as calculating the index of dissimilarity for very small groups leads to artificially high values.

The *second* step in the analysis was to apply the counterfactual method by comparing the observed change in residential segregation between the immigrant‐background group and the Finnish‐background group to a counterfactual situation in which the events related to the given population‐change process did *not* occur among the immigrant‐background group during the time interval under analysis (and similarly regarding these events among the Finnish‐background group). 4Bailey (2012) and Bailey et al. (2016) had the opposite design: They allowed only one process to occur in the counterfactual situation. Our rationale for our choice is that we only made a minimal change to the observed dynamics. Wessel et al. (2018) had the same approach. However, we report also how the results changed with the alternative design. For example, in the case of intraregional migration, the counterfactual situation was created by keeping the intraregional migrants in their *t*_0_ zip‐code areas at *t*_1_, and in the case of natural change, the counterfactual situation was obtained by keeping those who had died in their *t*_0_ neighbourhoods at *t*_1_ and removing the births at *t*_1_. 5We did not attempt to take into account the dynamic consequences of such counterfactual situations in terms of their effect on the migration of other groups besides the group in question, for example.


Segregation indices were calculated for *t*_1_ in the counterfactual scenarios, in which one demographic process between *t*_0_ and *t*_1_ among the immigrant‐background group or among the Finnish‐background group was removed at a time, and these values were then compared with the observed values. These comparisons roughly indicate the relative importance of different processes, especially the direction of their contributions. The main advantage of the counterfactual method is that it aims to show the net effects of different processes on segregation at the regional level. We could also use both the index of dissimilarity and the exposure index. However, there is more uncertainty in the results than in the other methods applied here, as the method only takes into account “first‐order” changes in population distributions without attempting to assess the interdependencies between different demographic processes.

The *final* step in the analysis was to calculate the contributions of different population change processes to the changes in the ethnic composition of particular types of neighbourhoods. This was done in order to illustrate how the findings concerning the indices are actually seen at the neighbourhood level. We divided the zip‐code areas into (population‐weighted) quintiles of the percentage of non‐Western‐origin population in the region at *t*_0_, and these quintiles were used as the neighbourhood types to be compared. Changes in each quintile were analysed using the design shown in Table 2, but in this case, changes in the numbers of persons were calculated instead of changes in exposure. This description was done using only the last period, from 2011 to 2014. In 2011, the percentage of non‐Western‐origin population varied in the highest quintile between 7.9% and 15.1% (number of zip‐code areas in this quintile = 29). In the lowest quintile, the percentage varied between 0% and 1.5% (*n* = 123).

The main advantages of the method in the third step are that it allowed us to directly decompose the population changes into the contributions of different processes and that it is based on observed changes. On the other hand, the method does not directly show the effects at the regional level but relates instead to specific types of neighbourhoods separately. Together, the three methods balance each other's shortcomings, so conclusions based on the findings from all three steps give the most complete overview.

## 4 RESULTS

### 4.1 Components of population change in the study region

The growth rate of the population in the Helsinki region varied during the 3‐year periods between 3.2% and 3.8% (in 3 years). The increase was much faster for the population with a non‐Western origin: In this population, the growth varied between 26.6% and 30.6%, whereas in the Finnish‐origin population, it varied between 1.0% and 1.4%. In the Finnish‐origin population, most of the growth came from positive natural change and, to a lesser extent, from internal migration from other regions of Finland. In the non‐Western‐origin population, the main contribution was from direct immigration to the region, while natural change had the next largest contribution.

### 4.2 Observed development of ethnic residential segregation

Between 2005 and 2014, the variation between zip‐code areas in the percentage of non‐Western‐origin population increased considerably in relative terms. The (population‐weighted) average proportion of the population with a non‐Western immigrant background in the zip‐code areas in the Helsinki region increased from 3.1% to 5.9%, whereas the range changed from 0–10% to 0–18% and the *SD* from 2.4% to 4.3%. 6In the city of Helsinki, the average changed from 4.1% to 7.5%, the range from 0–10.3% to 0–16.0%, and the *SD* from 2.5% to 4.2%. Table 3 shows the observed development of the index of dissimilarity and the isolation index between 2005 and 2014 in the region. In the case of the index of dissimilarity, the residential distribution of the non‐Western‐origin population is compared with that of the Finnish‐born population, whereas the isolation index is also affected by the shares of other immigrants than the non‐Western‐origin group.

**Table 3:** Development of the dissimilarity and isolation indices in the Helsinki region, for the population with a non‐Western immigrant background, 2005–2014

Segregation index	2005	2006	2007	2008	2009	2010	2011	2012	2013	2014
Index of dissimilarity	33.4	33.4	33.9	34.1	33.9	33.8	33.5	33.6	33.8	33.9
Isolation index	4.9	5.3	5.7	6.2	6.6	6.9	7.3	7.8	8.3	8.9

Source: Authors' own calculations, based on data from Statistics Finland.

The index of dissimilarity remained at the same level of 33–34% throughout the years under study. The interpretation is that 33–34% of either the Finnish‐born or non‐Western‐origin group should relocate to a different zip‐code area in order to achieve the same residential distribution in both groups. The isolation index, in turn, has been increasing steadily, reflecting the increasing immigrant population in the region. It shows that in 2005, the average share of the non‐Western‐origin population in the zip‐code areas where the non‐Western‐origin population lived was approximately 5%, and it increased to 9% in 2014. This rapid increase of the isolation index illustrates the “increased visibility” aspect of the development, which is hidden when using the index of dissimilarity. In situations characterised by a growing immigrant‐origin population, their share of the overall neighbourhood population can increase rapidly, even if the residential separation of the immigrant‐origin group from the ethnic majority is not increasing.

### 4.3 Changes in exposure to the non‐Western‐origin population

Table 4 shows how exposure to the non‐Western‐origin population differed between newcomers to the region and those who left the region. This is shown separately for the non‐Western‐origin and Finnish‐origin populations, by population change category for each population. In the case of stayers and intraregional migrants, the values show how different their zip‐code areas were at *t*_1_ in terms of exposure to the non‐Western‐origin population as compared with their *t*_0_ zip‐code areas. The total change values indicate the changes in the value of the isolation index, that is, changes in the exposure of the non‐Western‐origin group to members of the group, and changes in the total exposure of the Finnish‐origin population to the non‐Western‐origin population.

**Table 4:** Changes during each period in exposure to the total non‐Western‐origin population by population change category among the non‐Western‐origin and Finnish‐origin populations, percentage points

Population change category	Non‐Western‐origin population			Finnish‐origin population		
	Helsinki region					
	2005–2008	2008–2011	2011–2014	2005–2008	2008–2011	2011–2014
Total change	1.24	1.14	1.59	0.73	0.80	0.99
Stayers	1.28	1.26	1.66	0.76	0.82	1.01
Intraregional migrants	1.14	0.96	1.29	0.57	0.66	0.87
Between‐region migrants	2.23	1.49	2.23	1.19	1.29	1.48
International migrants	1.22	1.40	2.14	0.54	0.70	0.85
Natural change (births and deaths)	1.88	1.50	1.79	0.43	0.32	0.42
To/from household population	1.46	0.93	1.68	1.08	1.32	1.38

*Note*. The values show the changes in exposure to the non‐Western‐origin population within the given population‐change category among the country‐of‐origin group, that is, changes in the weighted average share of non‐Western‐origin population in the zip‐code area populations, with the weights being the number of people in the corresponding population‐change category of the country‐of‐origin group living in each zip‐code area. All periods start at the end of the first year and end in the end of the last year.

Starting from the non‐Western‐origin population, we can see that among those who were already living in the region at *t*_0_, exposure to the total non‐Western‐origin population increased less among the intraregional migrants than among the stayers. This suggests that intraregional migration among the non‐Western‐origin population *decreased* its spatial concentration. 7These outcomes are also affected by the other population‐change processes in all ethnic groups. Therefore, the observed outcomes tell not only about the given processes but also about what else has occurred in the areas between *t*_0_ and t_1_. In this case, the stayers are more exposed to new immigrants, for example. Of all migrant types, between‐region migrants have the largest differences between out‐migrants and in‐migrants: In‐migrants moved to zip‐code areas with clearly higher percentages of non‐Western‐origin populations than the between‐region out‐migrants had been living in. When considering natural change, shares of the non‐Western‐origin population have been higher where births have occurred than in the case of (the very few) deaths.

Also among the Finnish‐origin population, exposure to the non‐Western‐origin population increased less among the intraregional migrants than among the stayers. In this case, this suggests an *increasing* effect on ethnic segregation. Of all migrant groups, the difference between out‐movers and in‐movers was again the largest among the between‐region migrants, with in‐migrants moving to areas with higher shares of non‐Western‐origin population. Births among the Finnish‐origin population have occurred in areas with only slightly higher percentages of a non‐Western‐origin population than in areas where those who died during the period had been living 3 years earlier, suggesting that natural change has shifted the Finnish‐origin population to areas with less non‐Western‐origin residents.

The effects of the changes in exposure shown in Table 3 on the segregation between the non‐Western‐origin population and the Finnish‐origin population cannot be directly seen from this analysis. This is because the sizes of the population change components differed in terms of the number of persons, and the sizes also changed between *t*_0_ and *t*_1_ (see Table A1). For example, deaths in the non‐Western‐origin group have been so rare that the effect of natural change mostly depends on the spatial distribution of births. The counterfactual analyses, shown next, take these aspects into account.

### 4.4 Counterfactual scenarios

Our second method of assessing the significance of different population‐change processes on the segregation dynamics involved comparing the observed values of the segregation indices at *t*_1_ (end of each period) with values obtained for counterfactual scenarios. In each scenario, one population‐change process did not occur (see Section 3.2). We subtracted counterfactual values at *t*_1_ from the observed values, so the resulting values could be interpreted to indicate how much each demographic process had increased or decreased segregation, as measured by the index, during each period. We excluded moves to and from the household population from this analysis because their impact was negligible.

**Figure 1:**
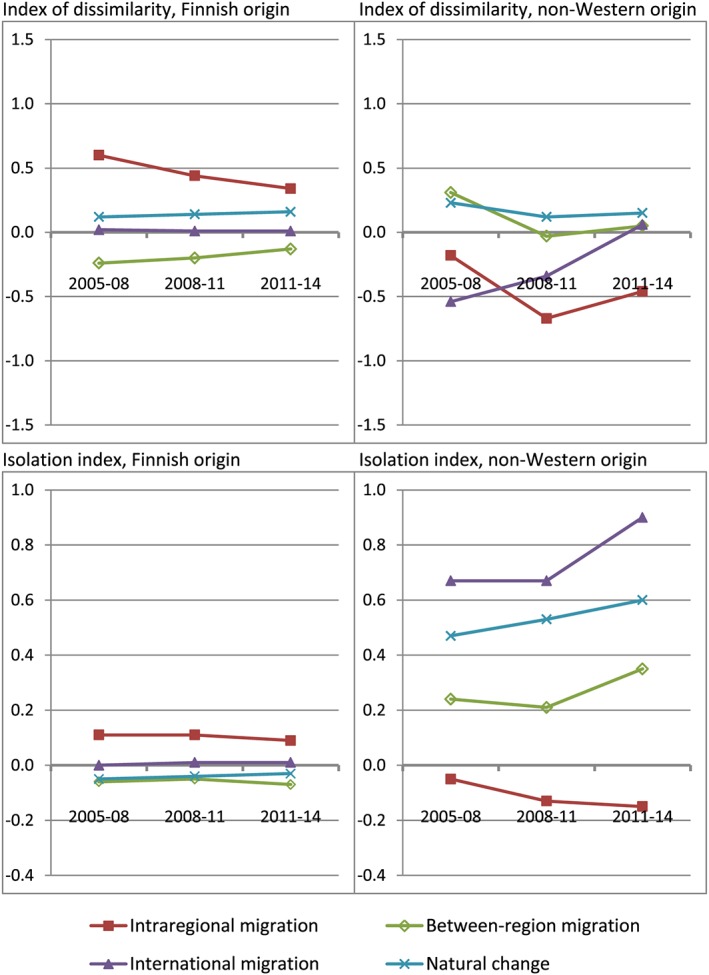
Contribution of each demographic process to the change of segregation indices between *t*_0_ and *t*_1_, based on counterfactual analysis (index value at *t*_1_ without the process = 0), by period, Helsinki region

Results are shown in Figure 1. Values on the *y* axis are on the (0–100) scale of each index. Negative values indicate that the corresponding population‐change process *decreased* segregation (the observed index value at *t*_1_ was that much lower than without the process). Similarly, positive values suggest that the process *increased* the level of the index. Therefore, we can see that in the case of the index of dissimilarity, intraregional migration among the non‐Western‐origin group decreased segregation, whereas intraregional migration among the Finnish‐origin population increased segregation. The other processes had smaller effects. Immigration of non‐Western immigrants seems to have decreased segregation during the first two periods, but not during the last period. The minor impacts of between‐region migration and natural change suggest that even though the spatial distributions differed for example between in‐migrants and out‐migrants (Table 4), the dominating flows, that is, in‐migration and births, did not visibly change the group's spatial distribution.

In the case of the isolation index, the findings concerning intraregional migration were similar to those obtained with the index of dissimilarity. However, the main processes contributing to increasing values were the direct immigration of non‐Western‐origin immigrants to the region and natural change within this group (excess number of births over deaths). The difference between these results and the corresponding observations regarding opposite or minor effects in the case of the index of dissimilarity again illustrates that processes increasing the immigrant population in the region may make large contributions to changes in the isolation index—and to the “visibility” of immigrants—even if they do not increase the residential separation between the non‐Western‐origin and Finnish‐origin populations. 8The sums of the effects of the individual processes cannot be expected to be exactly the same as the total observed changes in the indices, especially in the case of the index of dissimilarity. However, the sums (including the effects of moves to and from the household population) were quite close to the observed changes, and they indicate changes to the indices in the same directions as the observed changes. The largest difference in the case of *D* was 0.30, and the largest difference in the case of the isolation index was 0.12.


As a robustness analysis, we also created the counterfactual situations similarly as Bailey (2012), that is, by letting only one process occur at a time (above, we *omitted* one process at a time). The results were quite similar in this design as compared with those presented in Figure 1. However, the segregation‐increasing impact of intraregional migration among the Finnish‐origin population appears a little stronger in the alternative design, and the deconcentrating trend in the intraregional migration among the non‐Western‐origin population appears a little weaker than above.

### 4.5 Decomposition of population changes in zip‐code areas with high and low shares of non‐Western‐origin population

In our final analysis, we decomposed the population changes between 2011 and 2014 in the zip‐code areas on the basis of the percentage of non‐Western‐origin population in the zip‐code areas in 2011. We divided the zip‐code areas into (population‐weighted) quintiles in the region according to this percentage.

**Figure 2:**
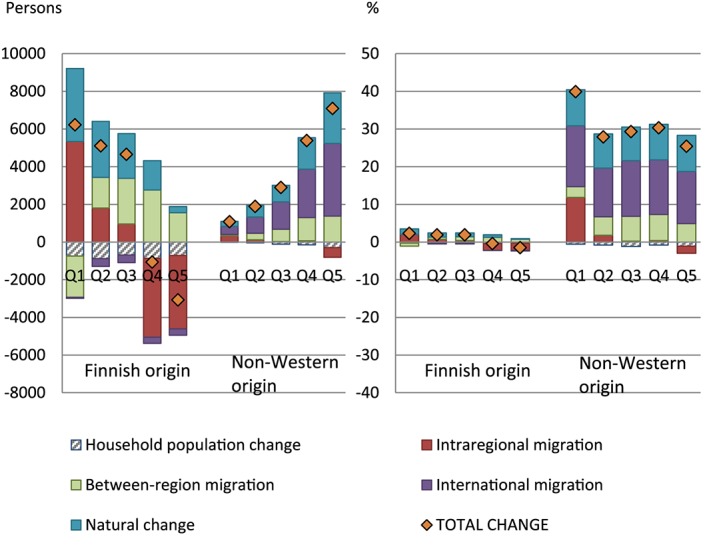
Changes in the Finnish‐origin and non‐Western origin populations in zip‐code areas by the quintile of the share of non‐Western‐origin population in 2011 (Q1 = lowest quintile, Q5 = highest quintile), Helsinki region 2011–2014, absolute numbers of people and percentages of 2011 populations

Figure 2 shows that an increase in the size of the non‐Western‐origin population in zip‐code areas where its share was highest was mostly due to positive international migration among this group. Also, natural growth and positive between‐region migration increased the group's size in these areas. Intraregional migration dispersed non‐Western‐origin population away from them. The Finnish‐origin population decreased in these areas mostly due to negative intraregional migration. The main process increasing the Finnish‐origin population in such areas was between‐region migration. In areas with the lowest share of non‐Western‐origin residents in 2011, the Finnish‐origin population grew mainly due to positive intraregional migration and also due to natural growth, while there was population loss due to between‐region migration. Likewise, the immigrant populations grew in these areas due to international migration and natural change, just as at the other end of the zip‐code area distribution, but also due to positive intraregional migration.

Across all areas, the non‐Western‐origin population increased in *absolute* numbers more in areas where their share was higher in 2011, whereas the Finnish‐origin population increased only in areas with intermediate or low percentages of non‐Western immigrants. *Relative* increase of the non‐Western‐origin population was highest in the zip‐code areas where their share of the population had been the lowest, and this difference to the other quintiles was mostly because of positive intraregional migration. The equal relative increases in the non‐Western‐origin population due to international migration and natural change across the quintiles explain the minimal impact of these processes on the index of dissimilarity observed above.

## 5 DISCUSSION

This paper investigated how different demographic processes of population change contribute to the development of ethnic segregation in the Helsinki region in Finland. We used three different methodological approaches to better understand the role of the different processes. Unlike previous studies, we focused on the *aggregate* region‐level impacts of demographic processes, for both the immigrant‐origin and native‐origin populations. All of our analyses indicate that in the Helsinki region, intraregional migration of the Finnish‐origin population increased ethnic segregation, whereas the intraregional migration of the non‐Western‐origin population decreased segregation.

Previous studies on the Netherlands (Musterd & de Vos, 2007; Zwiers et al., 2017) and Sweden (Bråmå, 2008) have also found that the residential mobility of immigrants leads to deconcentration, whereas a Norwegian study (Wessel et al., 2018) did not find such an impact. Zwiers et al. (2017) partly attributed the deconcentrating trend to the Dutch policy of urban restructuring, which has led to the replacing of lower income residents in immigrant concentration neighbourhoods with middle‐class native Dutch in‐movers. However, the present study and Bråmå (2008) have also found deconcentrating processes in other types of contexts. Similar to Musterd and de Vos (2007), Bråmå (2008), and Vilkama (2011), we conclude that migration of the native‐origin population can play a significant role in the production of immigrant concentrations. Our contribution to this research was to combine the different perspectives and approaches from these studies into one framework.

Regarding the other demographic processes, international migration to and from the region has decreased segregation or it has not had an impact, depending on the period. Natural change and between‐region migration in both Finnish‐origin and non‐Western‐origin populations have had only minor impacts on segregation. These findings do not contradict earlier observations (Bråmå, 2008; Finney & Simpson, 2009; Musterd & de Vos, 2007; Wessel et al., 2018) regarding the importance of natural change and immigration for the growth of ethnic minority concentrations. These processes have contributed to rising shares of non‐Western‐origin immigrants in zip‐code areas in the Helsinki region as well. But natural change and immigration have not directly increased the dissimilarity of residential distributions between the non‐Western‐origin population and the Finnish‐origin population in the Helsinki region, as their relative impacts have been similar in neighbourhoods with lower and higher shares of non‐Western‐origin population. Therefore, our results remind us that the increasing exposure of immigrants to each other does not necessarily mean increasing residential separation from the ethnic majority. On the other hand, the overall increase in non‐Western‐origin immigrants may have made segregation more visible, therefore increasing the importance of existing concentrations (see Enos, 2017). Increasing shares of immigrants in neighbourhood populations may be relevant also from the point of view of organising local services, even if the evenness of the group's spatial distribution, as measured by the index of dissimilarity, does not change. For these reasons, it is important that we not only look at the evenness of the residential distributions and intraregional mobility as a mechanism when assessing the dynamics of ethnic segregation. By measuring both the evenness and exposure dimensions of segregation, and by taking into account multiple demographic processes of change, a more complete picture can be drawn.

This analysis was designed to primarily offer a region‐level illustration of the significance of different demographic processes for the development of ethnic segregation. Analyses at the neighbourhood level, which were only touched upon here, are a natural next step in the analysis. Also, the counterfactual method applied in this study could be refined to address interdependencies between the different demographic processes.

An important aspect of ethnic segregation is its connection with socio‐economic segregation. Ethnic segregation may be considered problematic especially if the most “immigrant‐dense” neighbourhoods are also the poorest. This connection may change even if ethnic segregation does not change: For example, in Sweden, immigrant‐dense neighbourhoods became poorer between 1990 and 2010, despite stable levels of ethnic segregation, due to increasing income inequality (Hedman & Andersson, 2015). In their analysis of the Amsterdam region in the Netherlands, van Gent and Musterd (2016) found differences between migrants and the native Dutch population in the contributions of different socio‐demographic processes to population change. They concluded (p. 909) that the “newly forming social geography can no longer be understood by looking at migrants and social‐economic groups separately, or by conflating migrant groups and low‐income groups.” Therefore, a more complete understanding of the dynamics of segregation will require a combined analysis of ethnic and socio‐economic segregation. Such an analysis may be more easily used to inform policymakers on the implications of the observed development of ethnic segregation.

The main policy‐related outcome of the present analysis is that there is no indication of a general tendency of self‐segregation among the non‐Western‐origin population. This means that at least concerning the total non‐Western‐origin population in the Helsinki region, policies aiming at the cultural integration of immigrants would not directly address the main process increasing ethnic segregation, which is the selective intraregional migration among the Finnish‐origin population. This migration may be related to other characteristics of neighbourhoods besides their ethnic composition, such as social problems, safety, and the reputation of the neighbourhood (Vilkama, Vaattovaara, & Dhalmann, 2013), which can all be addressed with other types of interventions.

## APPENDIX A

**Table A1:** Distributions of the non‐Western‐origin population into population change categories by period

		2005–2008	2008–2011	2011–2014
*t*_0_	Stayer	25,020	32,754	40,773
	Intraregional out‐mover	10,115	13,525	17,818
	Between‐region out‐mover	741	1,147	1,293
	Emigrant	2,095	2,204	2,851
	Death	52	95	130
	From household population	1,340	1,689	2,208
	Total *t*_0_	39,363	51,414	65,073
*t*_1_	Stayer	25,020	32,754	40,773
	Intraregional in‐mover	10,115	13,525	17,818
	Between‐region in‐mover	2,517	3,198	4,953
	Immigrant	8,857	9,323	12,049
	Birth	4,203	5,222	6,227
	To household population	702	1,051	1,581
	Total *t*_1_	51,414	65,073	83,401
